# Effects of Contributor Experience on the Quality of Health-Related Wikipedia Articles

**DOI:** 10.2196/jmir.9683

**Published:** 2018-05-10

**Authors:** Peter Holtz, Besnik Fetahu, Joachim Kimmerle

**Affiliations:** ^1^ IWM Leibniz-Institut für Wissensmedien (Knowledge Media Research Center) Knowledge Construction Lab Tübingen Germany; ^2^ L3S Research Center Leibniz University of Hannover Hannover Germany

**Keywords:** Wikipedia, health-information online, collaborative knowledge construction, contributor characteristics

## Abstract

**Background:**

Consulting the Internet for health-related information is a common and widespread phenomenon, and Wikipedia is arguably one of the most important resources for health-related information. Therefore, it is relevant to identify factors that have an impact on the quality of health-related Wikipedia articles.

**Objective:**

In our study we have hypothesized a positive effect of contributor experience on the quality of health-related Wikipedia articles.

**Methods:**

We mined the edit history of all (as of February 2017) 18,805 articles that were listed in the categories on the portal health & fitness in the English language version of Wikipedia. We identified tags within the articles’ edit histories, which indicated potential issues with regard to the respective article’s quality or neutrality. Of all of the sampled articles, 99 (99/18,805, 0.53%) articles had at some point received at least one such tag. In our analysis we only considered those articles with a minimum of 10 edits (10,265 articles in total; 96 tagged articles, 0.94%). Additionally, to test our hypothesis, we constructed contributor profiles, where a profile consisted of all the articles edited by a contributor and the corresponding number of edits contributed. We did not differentiate between rollbacks and edits with novel content.

**Results:**

Nonparametric Mann-Whitney U-tests indicated a higher number of previously edited articles for editors of the nontagged articles (mean rank tagged 2348.23, mean rank nontagged 5159.29; U=9.25, *P*<.001). However, we did not find a significant difference for the contributors’ total number of edits (mean rank tagged 4872.85, mean rank nontagged 5135.48; U=0.87, *P*=.39). Using logistic regression analysis with the respective article’s number of edits and number of editors as covariates, only the number of edited articles yielded a significant effect on the article’s status as tagged versus nontagged (dummy-coded; Nagelkerke *R^2^* for the full model=.17; *B* [SE *B*]=-0.001 [0.00]; Wald *c^2^* [1]=19.70; *P*<.001), whereas we again found no significant effect for the mere number of edits (Nagelkerke *R^2^* for the full model=.15; *B* [SE *B*]=0.000 [0.01]; Wald *c^2^* [1]=0.01; *P*=.94).

**Conclusions:**

Our findings indicate an effect of contributor experience on the quality of health-related Wikipedia articles. However, only the number of previously edited articles was a predictor of the articles’ quality but not the mere volume of edits. More research is needed to disentangle the different aspects of contributor experience. We have discussed the implications of our findings with respect to ensuring the quality of health-related information in collaborative knowledge-building platforms.

## Introduction

### Health Information Online

Discussions regarding the quality of health-related information on the internet go back as far as the late 1990s [1,2] and have continued recently [3-5]. Consulting the internet for health-related information has undoubtedly become a common and widespread phenomenon [6,7]. Over the last several years, Wikipedia has emerged as one of the most important knowledge resources for health-related information on the Web [4,8,9]. In this paper we (1) describe potential quality issues as indicated by community-applied tags in health-related Wikipedia articles, and (2) analyze the importance of contributor experience for the quality of health-related Wikipedia articles.

### Wikipedia as a Resource

Wikipedia relies heavily on peer review to ensure the quality of its collaboratively constructed knowledge corpus, and Wikipedia contributors are expected and invited to correct other contributors’ mistakes [9]. Controversial and conflictual issues are to be debated by the contributors until consensus is reached [10,11]. One way of instigating such a discussion is to apply one of several Wikipedia template messages (or *tags*) to an article, which indicate quality-related problems, such as articles being biased, misleading, or factually wrong. In our study, we used the occurrence of at least one quality-issue tag in an article’s edit history as a proxy for potential quality problems.

Although Wikipedia is not governed by a body of experts, the quality of health-related Wikipedia articles is not necessarily worse than that of expert-generated internet content [12]. There have been repeated calls for experienced medical professionals to get more actively involved in improving the accuracy of health-related Wikipedia articles [13,14]. Nevertheless, there is empirical evidence that, in spite of all efforts to ensure knowledge quality, Wikipedia articles can be biased; for example, as a consequence of predominantly male Wikipedia authors underreporting or belittling notable achievements of women (*gender bias*) [15]. Another form of bias is the presentation of information regarding Wikipedia authors’ own national group appearing in a more positive way than information about other groups (*ingroup bias*) [16]. In line with previous research [17,18], we hypothesize that a certain proportion of such distortions is attributable to a lack of experience on the side of the respective article’s contributors.

## Methods

In this study we mined the complete edit history (as of February 2017) of the 18,805 articles that were listed within the categories of the Wikipedia portal *health & fitness* [19] in the English language version of Wikipedia. We developed our own publicly available code to mine the data [20]. We first identified the occurrence of tags or template messages within the articles’ edit histories that indicated quality issues. These tags were *neutral point of view policy violation* (42 cases in our sample), *contradictory content* (13), *unbalanced content* (12), *confusing content* (17), and *inaccurate content* (23). Tags in the articles’ respective talk pages were not taken into account. Tags such as *neutral point of view policy violation* and *unbalanced content* refer to violations of Wikipedia’s standards of objectivity, which may be caused by social biases such as an ingroup bias or a gender bias, while the other tags are related to quality issues in general. Of all the sampled articles, 99 (99/18,805, 0.53%) articles had at some point received at least one of the aforementioned tags. For the comparison of tagged versus nontagged articles, we only used articles that were comprised of at least 10 edits, although it is imaginable that some elaborated Wikipedia articles result from relatively few comparatively substantial edits. This limitation reduced the total number of articles to 10,265, whereas the total number of occurrences of tagged articles was reduced from 99 to 96 (96/10,265, 0.94%).

For every contributor that authored at least one edit within the sampled articles, we constructed the contributor’s profile, consisting of the total number of edits in all Wikipedia articles and the total number of articles that the contributor had edited up to that point. We did not make any distinction with respect to the quality of edits; hence, we treated rollbacks and edits with novel content in the same way. For all statistical analyses, we used the SPSS 22 software package. All reported significance tests are two-sided and we set the significance level at *P*=.01.

## Results

### The Content of the Tagged Articles

The 99 tagged articles were manually assigned to one of four different content categories that had emerged in the analysis: *legislation & politics* (41 articles; examples included “abortion in Iran,” “free market healthcare,” and “smoking ban”), *medicine-related topics* (21 articles; examples included “antimicrobial resistance,” “obesity,” and “zidovudine”), *alternative medicine-related topics* (19 articles; examples included “astrology and health,” “chiropractic,” and “siddha medicine”), *and places, people, and events* (18 articles; examples included “2009 flu pandemic in Mexico,” “Bethlem Royal Hospital,” and “Arnold Schwarzenegger”). For details see Multimedia Appendix 1.

The average number of total edits for these 99 articles was 940.60 (SD 1458.67), and the articles were authored by an average of 186.27 (SD 251.97) individual contributors. In comparison to the 18,706 nontagged articles (mean edits 49.67, SD 194.56; mean editors 16.16, SD 38.26), the tagged articles were comprised of a significantly higher number of edits (*t*_98.02_=6.10; *P*<.001; *d*=1.08), and they were authored by a significantly higher number of contributors (*t*_98.02_=6.72; *P*<.001; *d*=1.17). Part of these differences can be explained by the fact that a substantial number of the nontagged articles were “stubs” which featured nothing more than a mere article title. Such stubs (fewer than ten edits) were omitted from all further analyses. As a consequence, the differences between the tagged (mean edits 969.75, SD 1472.23; mean editors 191.96, SD 253.81) and the nontagged articles (mean edits 88.13, SD 257.66; mean editors 27.30, SD 49.16) comprising 10 or more edits with regard to the average total numbers of edits (*t*_95.06_=5.87, *P*<.001, *d*=1.07) and editors (*t*_95.07_=6.36, *P*<.001, *d*=1.09) could be reduced to some extent, but the difference still remained significant.

### Effects of Contributor Experience

The 10,265 remaining articles had an average of 100.12 edits (SD 311.00) and were authored by an average of 29.79 (SD 57.97) editors. According to their user profiles, the editors of these articles had made on average 32,031.05 (SD 27,513.01) edits in 1,033.42 (SD 648.71) Wikipedia articles. The number of the editors’ total edits, as well as the number of edited articles, were positively skewed and were not normally distributed according to Kolmogorov-Smirnov tests (*P* values <.001). Hence, we used Mann-Whitney U-tests to analyze differences between the authors of the 96 articles that were comprised of at least 10 edits, and that received at least one of the tags indicating quality issues, and those of the nontagged articles with regard to the editors’ previous editing activities. We found a significant difference with regard to the total number of edited articles (mean rank tagged 2348.23, mean rank nontagged 5159.29; *U*=9.25, *P*<.001) whereas the difference in terms of the total number of edits did not reach statistical significance (mean rank tagged 4872.85, mean rank nontagged 5135.48; *U*=0.87, *P*=.39).

To account for the significant differences between the tagged and the nontagged articles comprising 10 or more edits with regard to the average total numbers of edits and editors (see above), we further tested our initial findings using logistic regression analyses (stepwise) with the tagged versus nontagged status (dummy coded) of the articles as the dependent variable, the respective article’s numbers of total editors and total edits as control variables, and the contributor’s total number of edits and edited articles (respectively) as independent predictors. This approach controlled for a possible obfuscating linear effect of the number of an article’s edits or editors. Again we found that the total number of articles that were edited by the contributors significantly predicted the articles’ statuses (Nagelkerke *R*^2^ for the full model=.17; *B* [SE *B*]=-0.001 [0.00]; Wald *c*^2^[1]=19.70; *P*<.001), whereas the total number of the contributors’ edits did not yield a significant effect (Nagelkerke *R*^2^ for the full model=.15; *B* [SE *B*]=0.000 [0.01]; Wald *c*^2^[1]=0.01; *P*=.94).

## Discussion

### Principal Results

The largest category of health-related Wikipedia articles that had at one point in their edit history received a user-applied tag indicating quality issues focused on topics about political and legislative issues. The other articles were related to alternative medicine, generic medical topics, and specific events and people.

The authors of the tagged articles had (on average) edited less Wikipedia articles than the authors of the nontagged articles. However, we did not find a significant difference for the mere number of the contributor’s previous edits. Assuming that there is a relationship between the authors’ competence and the probability that a Wikipedia article receives a tag indicating quality issues, this could indicate that the mere volume of activity is not indicative of a Wikipedia author’s competence, but rather a certain breadth of experience. This finding, if corroborated, could have implications for Wikipedia’s (and other platforms’) editing system as well as for the quality management of collaborative knowledge construction platforms. For example, articles that were authored by editors with a comparatively narrow range of previously edited articles could be automatically identified and marked for further quality checks as a means of ensuring and improving the quality of health-related articles. However, more research is needed to disentangle the effects of the different facets of Wikipedia contributor activities on the quality of Wikipedia articles.

### Limitations

A major limiting factor for our study was that relatively few articles received at least one of the tags indicating quality issues. One way of addressing this issue in future studies, to replicate our findings, would be to use quality metrics that are based on article features such as length, the number of paragraphs, and the number of pictures [21]. It should also be noted that the authors of both the tagged and the nontagged articles had (on average) made several thousand edits in several hundred articles, and hence were relatively experienced Wikipedia contributors. Further research is needed to analyze the effects of different levels of editor experience (or inexperience) on the quality of Wikipedia articles. The articles that were sampled for this study only constitute a part of the medical content that is available at Wikipedia [22]. Future studies are needed to replicate our findings for a wider range of health-related Wikipedia articles and articles in languages other than English.

### Conclusion

Consistent with previous studies [4,13,21], our findings highlight the potential of Wikipedia as a valuable resource for health-related information. However, the quality of Wikipedia articles relies on the willingness of experienced and knowledgeable contributors to take on the unpaid labor of editing and improving Wikipedia articles. One way of encouraging experienced professionals to further engage with Wikipedia content would be to provide incentives for such activities (eg, in the form of continuing medical education credits) [22].

## Appendix

Multimedia Appendix 1 Overview of the tagged articles.
