# Supplementary material for: Effects of Contributor Experience on the Quality of Health-Related Wikipedia Articles
Source: J Med Internet Res. 2018 May 10;20(5):e171. doi: 10.2196/jmir.9683 (PMC5968213; doi:10.2196/jmir.9683)
Supplement: Multimedia Appendix 1 [file jmir_v20i5e171_app1.pdf]

Multimedia Appendix 1

Overview of the tagged articles

**Title**	**Revisions**	**Editors**	**Tags**
**Legislation / Politics**			
Abortion in Iran	85	50	CONT
America's Affordable Health Choices Act of 2009	639	109	NPOV
Canada Health Act	243	74	NPOV
Cartwright Inquiry	195	47	INACC
Child health in Uganda	19	9	NPOV
Comparison of the healthcare systems in Canada and the United States	1840	321	NPOV
E-patient	126	46	NPOV
Electronic prescribing	164	49	NPOV
Emergency care assistant	98	24	UNBAL
Essential medicines policies	61	22	UNBAL
Evidence-based medicine	804	236	NPOV
Evidence-based practice	269	96	INACC
Free-market healthcare	138	46	NPOV
Health care in Israel	414	81	NPOV
Health care reform debate in the United States	976	172	NPOV, INACC (3)
Health care reform in the United States	3044	470	NPOV (5)
Health information management	180	67	CONF
Indian Health Service	194	77	INACC (4)
Jahi McMath case	233	25	INACC (2)
Joint Commission	427	107	CONT
Lenus the Irish Health Repository	11	5	CONF
Medical prescription	1107	292	CONT
Medical Rural Bonded Scholarship Scheme	92	20	UNBAL
National Health Service (England)	2081	493	NPOV
Order 81	23	10	INACC
Patient Protection and Affordable Care Act	6716	904	NPOV (2), CONT
Pharmacoeconomics	80	46	CONF
Planned Parenthood	3623	541	NPOV (4)
Poverty in the United States	2200	496	INACC
Pre-existing condition	204	48	CONF
Race and health in the United States	83	33	CONF
~~Saudi Commission for Health Specialties~~	~~9~~	~~6~~	~~NPOV~~
School-based health centers	46	20	CONF

Single-payer healthcare	1892	297	NPOV (2), CONF
Smoking ban	2607	475	NPOV (2)
Texas Medication Algorithm Project	97	32	NPOV
Therapeutic discovery project	11	11	CONF
United States Department of Health and Human Services	719	278	NPOV
United States Public Health Service	312	104	CONF
Universal health care	3947	595	NPOV, UNBAL
World Health Organization	2360	681	FALSCH
**Medicine-related topics**			
Antimicrobial resistance	1814	355	UNBAL
Asbestos	1433	353	NPOV (2)
Bovine somatotropin	1145	259	CONT
Cortisol	949	263	CONT
Dietitian	390	129	INACC (2)
Dopamine	1723	273	INACC
Human microbiota	451	149	CONF
Illness-Wellness Continuum	10	6	CONF
Nitrous oxide	1721	447	CONF
Obesity	5065	1133	INACC
Obesity in Brazil	12	6	UNBAL
Obesity in Canada	111	42	INACC
Obesity in Mexico	96	38	INACC
Passive smoking	1636	239	NPOV, INACC
Placenta praevia	128	50	CONT
Potassium iodide	542	156	CONF
Prussian blue	297	108	INACC
Suboptimal health	46	17	INACC
Sunburn	850	242	CONT
Sunscreen	179	32	INACC (2)
Zidovudine	643	159	UNBAL, INACC
**Alternative-medicine-related topics**			
~~Astrology and health~~	~~5~~	~~3~~	~~NPOV~~
Autism Awareness Campaign UK	220	52	NPOV
Ayurveda	2294	411	NPOV
Bally Total Fitness	311	94	NPOV
~~Barefoot movement~~	~~9~~	~~4~~	~~UNBAL~~
Chiropractic	6874	649	NPOV (6), INACC
Chiropractic controversy and criticism	642	102	NPOV

Information therapy	43	29	CONF
List of traditional Chinese medicines	250	32	CONF (2)
Love Canal	874	230	NPOV
Mami Wata	689	114	INACC (2)
MindFreedom International	190	64	NPOV
Neurodiversity	348	71	UNBAL
New Century Health	50	21	NPOV
Osteopathic medicine	27	16	NPOV
Psychiatric survivors movement	237	66	NPOV
Siddha medicine	234	82	NPOV
The China Study	1000	184	UNBAL
Unani medicine	474	158	CONF
**Places / People / Events**			
2009 flu pandemic in Mexico	321	130	CONT
2009 flu pandemic timeline	4009	274	CONT
ALZA	52	13	INACC
Arnold Schwarzenegger	2584	712	INACC
Bethlem Royal Hospital	1526	175	NPOV
Chernobyl disaster	6433	1450	CONT, INACC
Christine K. Cassel	52	22	NPOV
Derzelas	117	31	INACC
Imhotep	1297	324	CONT
International No Diet Day	305	70	UNBAL
Kool Smiles	106	20	NPOV
Marcus de Laune Faunce	25	5	NPOV
Optometry in Singapore	127	27	NPOV
Osiris	3448	699	CONT
Robert J. White	206	80	NPOV
St. Mary's/Duluth Clinic Health System	55	19	NPOV
Vielife	18	12	NPOV
Winthrop University Hospital	57	25	CONF

Note. NPOV = neutral point of view policy violation; CONT = contradictory content; CONF = confusing content; INACC = inaccurate content; UNBAL = unbalanced content. Crossed out articles were omitted for further analysis because the number of edits was smaller than 10.
